# A comprehensive study on the relationship between structure and antioxidant activity of natural deep eutectic solvent extracted polysaccharides from *Chinese yam* peel using asymmetrical flow field-flow fractionation

**DOI:** 10.1016/j.fochx.2026.104167

**Published:** 2026-07-02

**Authors:** Yao Huang, Liu Yang, Tinghui Yin, Mu Wang, Siyu Wang, Weiming Wang, Haiyang Dou

**Affiliations:** a Key Laboratory of Pathogenesis Mechanism and Control of Inflammatory-Autoimmune Disease of Hebei Province, School of Basic Medical Sciences, Hebei University, Baoding 071000, China; b Department of Gynecology, Affiliated Hospital of Hebei University, Baoding 071000, China; c State Key Laboratory of New Pharmaceutical Preparations and Excipients, Hebei University, Baoding 071002, China; d Key Laboratory of Medicinal Chemistry and Molecular Diagnosis of Ministry of Education, Hebei University, Baoding 071002, China

**Keywords:** *Chinese yam* peel polysaccharide, Natural deep eutectic solvent, Asymmetrical flow field-flow fractionation, Antioxidant

## Abstract

In this study, an ultrasound-assisted natural deep eutectic solvents (NADES) combined with enzymatic deproteinization was developed for extracting *Chinese Yam* peel polysaccharide (CYPP) from two varieties. The result demonstrated that the viscosity of NADES was the primary factor affecting the extraction rate of CYPP. Under optimized extraction conditions, the CYPP extraction rate obtained by NADES was 2.42-fold higher than that obtained by the water extraction method. Moreover, the relationship between the structure and antioxidant activity of CYPP was investigated using asymmetrical flow field-flow fractionation (AF4), Fourier transform infrared spectroscopy, and Congo red assay. The results suggested that NADES extraction method did not alter the functional groups and triple-helix structure of CYPP. Notably, the AF4 results suggested that NADES extracted CYPP had a relatively compact structure, which might play a crucial role in the antioxidant activities of CYPP than either its triple-helix structure or uronic acid content.

## Highlights

• CYPP was extracted and structurally characterized by NADES and AF4-MALS-dRI.
• Compared to water method, a 2.42-fold extraction rate of CYPP was obtained by NADES.
• The viscosity of NADES was the primary factor affecting the extraction rate of CYPP.
• The compact structure of CYPP plays a vital role in its antioxidant activity.

## 1 Introduction

*Chinese yam* (*Dioscoreae Rhizoma*) is the homology of food and medicine, and native to northern China (Guo et al., 2024). In the *Chinese yam* processing, *Chinese yam* peel is usually discarded as waste, which not only pollutes the environment but also wastes resources (Meng et al., 2019). However, *Chinese yam* peel contains a variety of bioactive components, including polysaccharides, flavonoids, and polyphenols (Liu et al., 2016). Among these, *Chinese yam* peel polysaccharide (CYPP) has attracted increasing attention due to its antioxidant, immunomodulatory, and hypoglycemic activities. Therefore, the in-depth development of *Chinese yam* peel offers a promising strategy for the comprehensive utilization of this crop and for environmental protection.

The extraction method significantly affects the structure and bioactivities of polysaccharides (Yu et al., 2025). The commonly used extraction methods for CYPP include water extraction, enzyme-assisted extraction, ultrasound-assisted extraction, and microwave-assisted extraction (Yang, Sun, et al., 2025). However, hot water extraction method is time-consuming and requires high temperatures, which can degrade polysaccharides and reduce their bioactivities (Xue & Farid, 2015). Although enzyme-assisted extraction can decompose the tissue structure of plant cells and accelerate the release of polysaccharides, strict extraction conditions are needed. Ultrasound-assisted extraction and microwave-assisted extraction methods can not only reduce extraction time and temperature, but also enhance extraction rate and bioactivity of polysaccharides (Guo et al., 2025). However, microwave may generate local high temperature that alter polysaccharide structure (Zhang, Lin, et al., 2024). Consequently, developing novel extraction methods for CYPP is of considerable importance.

Natural deep eutectic solvent (NADES) has emerged as a green solvent for polysaccharide extraction, owing to its low toxicity and sustainability (Gao et al., 2024). NADES is prepared by combining hydrogen bond donor (HBD) and hydrogen bond acceptor (HBA), which mainly consists of biological metabolites such as sugars, organic acids, amino acids, urea, and amines (Wang & Li, 2022). Unlike salts, cations in NADES compete with hydrogen atoms to bond with anions. Hydrogen bonding interaction among the components in NADESs is the primary driving force for the formation of eutectic system (Li, Yang, et al., 2025). NADESs can form strong hydrogen bonds with polysaccharides and disrupt polysaccharides-cell wall linkages, thereby enhancing extraction efficiency (Md Yusoff & Shafie, 2025). Moreover, their low melting point helps preserve polysaccharide bioactivity during the extraction process. Although the factors such as NADES composition, viscosity, polarity, and extraction conditions affect polysaccharide extraction rate (Wu et al., 2025), few studies have been reported on the application of NADES for CYPP extraction.

Beyond extraction, accurate structured characterization is essential for understanding polysaccharide structure-activity relationships. Plant polysaccharides often possess complex structures, ultrahigh molar mass (*M*_w_). It is well documented that in addition to monosaccharide composition and glycosidic linkages, the advanced structure and *M*_w_ distribution also affect the bioactivities of polysaccharides (Dai et al., 2021). It has been reported that *Chinese yam* polysaccharides are predominantly of the β-configuration, whereas CYPP contains a mixture of both α- and β-configurations (Bai et al., 2026). Chen et al. (2024) reported that highly branched conformation of polysaccharides may enhance their antioxidant capacity, whereas spherical, sheet, or honeycomb conformation with porous are also conducive to their antioxidant activity. However, the complexity and diversity of polysaccharides structures result in significant challenges to their separation and structural characterization. Size exclusion chromatography (SEC) is widely applied for the separation of polysaccharides. However, for the analysis of the sample with *M*_w_ generally larger than 10^6^ Da, SEC is limited due to the column adsorption and shear effect of the stationary phase. Furthermore, the free proteins and polysaccharide-binding proteins can interact with the SEC column packing material, thereby affecting the separation of polysaccharides (Li, Zhang, et al., 2025). Asymmetrical flow field-flow fractionation (AF4) has been proved to be a promising approach for the separation of polysaccharides due to the wide detection range in size (approximately from 1 nm up to 1 μm in the normal mode) and the utilization of ‘open channel’ without stationary phase or packing materials, which minimizes the column adsorption and shear degradation risks (Chen et al., 2021). Moreover, AF4 coupled online with ultraviolet-visible (UV), multiangle light scattering (MALS), and differential refractive index (dRI) detectors (AF4-UV-MALS-dRI) can provide the *M*_w_, radius of gyration (*R*_g_), apparent density (*ρ*_app_), and conformational information of polysaccharides (Guo et al., 2023). This approach has enabled structure-activity studies, e.g., revealing that a spherical conformation of a polysaccharide complex from *Tremella fuciformis* with stronger antioxidant activity (Chen et al., 2022). In this study, the main objectives were to develop an efficient NADES-based extraction method for CYPP and to investigate the relationship between the structure and antioxidant activity of CYPP derived from two Chinese yam varieties (*Xiaobaizui Chinese yam* denoted as XCYPP and *Tiegun Chinese yam* denoted as TCYPP) using AF4-UV-MALS-dRI.

## 2 Materials and methods

### 2.1 Materials and reagents

*Xiaobaizui Chinese yam* and *Tiegun Chinese yam* were purchased from a local supermarket (Baoding, China). They were confirmed as *Dioscorea opposite* Thunb. by Professor Shengci Fan (an expert in the field of Traditional Chinese Medicine at the College of Traditional Chinese Medicine, Hebei University). Choline chloride (ChCl), betaine (BET), citric acid (CA), malonic acid (MA), propylene glycol (PG), xylitol (Xyl), glycerol (Gly), L-Proline (Pro), fructose (Fru), urea, sodium hydroxide (NaOH), sodium nitrite (NaNO_2_), 1-phenyl-3-methyl-5-pyrazolone (PMP), trifluoroacetic acid (TFA), mannose (Man), ribose (Rib), rhamnose (Rha), glucuronic acid (GlcA), galacturonic acid (GalA), glucose (Glc), xylose (Xyl), galactose (Gal), arabinose (Ara), acetonitrile, methanol, potassium dihydrogen phosphate, and ethanol were purchased from Shanghai Macklin Biochemical Co., Ltd. (Shanghai, China). Chloroform was obtained from Damao Chemical Reagent Co., Ltd. (Tianjin, China). Hydrochloric acid (37%) and sulfuric acid were obtained from Beijing Chemical Co., Ltd. (Beijing, China). 3-Phenylphenol and D (+)-glucose were purchased from Shanghai Yuanye Bio-Technology Co., Ltd. (Shanghai, China). Alkaline proteinase, dispase, papain, 1,1-diphenyl-2-picrylhydrazyl (DPPH), phosphate-buffered saline (PBS), dimethyl sulfoxide (DMSO), trypsin, Dulbecco's modified eagle medium (DMEM), MTT cell proliferation and cytotoxicity assay kit, hydroxyl free radical scavenging capacity assay kit, ABTS^+^ free radical scavenging capacity assay kit, and total antioxidant capacity (T-AOC) assay kit were obtained from Beijing Solarbio Science & Technology Co., Ltd. (Beijing, China). Phenol was obtained from Shijiazhuang Organic Chemical Co., Ltd. (Shijiazhuang, China). Congo red was purchased from Shanghai Aladdin Biochemical Technology Co., Ltd. (Shanghai, China). Fetal bovine serum (FBS) was purchased from Hangzhou Sijiqing Bioengineering Materials Co., Ltd. (Hangzhou, China). Bovine serum albumin (BSA) was obtained from Sigma-Aldrich Co., Ltd. (Shanghai, China). Deionized water was obtained from a UPR-II-10 T Ultra-Pure water system from Sichuan ULUPURE Technology Co., Ltd. (Sichuan, China). All chemicals were of analytical grade and used without further purification.

### 2.2 Preparation and determination of physical properties of NADES

NADESs were prepared using a water bath heating method at different molar ratios of HBA (ChCl, BET, and Pro) to HBD (CA, MA, PG, Xyl, Urea, Gly, and Fru) as shown in Table 1. Briefly, the mixture of HBA and HBD was stirred in a water bath at 85 °C until a transparent liquid was formed. The pH value of NADES was measured using a PE28 pH meter (Mettler-Toledo International Inc., Zurich, Switzerland) at room temperature and the kinematic viscosity of NADES was determined using an Ubbelohde viscometer (Shanghai Liangjing Glassware Co., Ltd., Shanghai, China). The results are listed in Table 1. The viscosity of NADES was calculated by Eq. (1):

(1) V=c×t

where *V* represents the kinematic viscosity of the liquid, *c* is the instrument constant (0.003102 mm^2^/s^2^), and *t* is the elapsed time for the liquid level to traverse between the upper and lower calibration marks.

**Table 1:** Components and physical properties of natural deep eutectic solvents (NADES).

Type of NADES	HBA	HBD	Molar ratio	pH	Kinematic Viscosity (mm^2^/s)
NADES 1	ChCl	CA	1:1	−0.33 ± 0.01^p^	32.88 ± 0.47^c^
NADES 2		MA	1:2	−0.39 ± 0.00^q^	9.76 ± 0.17^j^
NADES 3		PG	1:2	7.19 ± 0.02^h^	9.68 ± 0.42^j^
NADES 4		Xyl	1:2	7.44 ± 0.02^g^	26.25 ± 0.67^f^
NADES 5		Urea	1:2	9.27 ± 0.01^b^	5.77 ± 0.65^k^
NADES 6		Gly	1:2	6.48 ± 0.02^i^	13.16 ± 0.56^i^
NADES 7		Fru	1:1	3.61 ± 0.02^l^	34.12 ± 0.39^b^
NADES 8	BET	CA	1:1	2.31 ± 0.00^m^	21.36 ± 0.44^h^
NADES 9		MA	1:2	2.03 ± 0.00^n^	22.56 ± 0.90^g^
NADES 10		PG	1:2	7.52 ± 0.01^f^	32.26 ± 0.54^c^
NADES 11		Xyl	1:2	7.97 ± 0.02^c^	30.17 ± 0.45^d^
NADES 12		Urea	1:2	9.75 ± 0.01^a^	5.44 ± 0.31^k^
NADES 13		Gly	1:2	7.72 ± 0.01^e^	36.59 ± 0.64^a^
NADES 14		Fru	1:1	5.64 ± 0.01^k^	28.36 ± 0.59^e^
NADES 15	Pro	Urea	1:2	6.00 ± 0.01^j^	9.99 ± 0.17^j^
NADES 16		Gly	1:2	7.87 ± 0.01^d^	28.74 ± 0.49^e^
NADES 17		MA	1:2	1.93 ± 0.00^o^	12.31 ± 0.37^i^

Note: The data are expressed as mean ± standard deviation (*n* = 3). Different superscript letters in the same column represent the statistical significance (*p*﹤0.05).

### 2.3 Optimization of NADES extraction parameters

*Chinese yam* peel was dried in a DHG-914385 electric thermostatic drying oven (Shanghai Xinmiao Medical Device Manufacturing Co., Ltd., Shanghai, China). The dried *Chinese yam* peel powder was crushed and defatted with 95% ethanol for 24 h to remove small lipophilic molecules. In this study, an ultrasound-assisted NADES extraction method was conducted to extract CYPP. Briefly, *Chinese yam* peel powder (1.0 g) and NADES were added to a 50 mL beaker and treated by a KQ5200DE ultrasound apparatus (Kunshan Ultrasound Instrument Co., Ltd., Jiangsu, China). The extract was centrifuged at 4000*g* for 10 min, and the supernatant was added with 95% ethanol to precipitate CYPP. After centrifugation, the precipitate was lyophilized by a freeze-dryer (SCIENTZ-10 N, Ningbo, China) to obtain crude CYPP. To obtain the maximum total saccharide extraction rate of CYPP, the extraction conditions were optimized via single-factor experiments (Wu et al., 2025). Taking the extraction rate and total saccharide content of CYPP as response values, six factors (i.e., NADES type, molar ratio of HBA to HBD (3:1, 2:1, 1:1, 1:2, 1:3, and 1:4), water content (10%, 20%, 30%, 40%, 50%, 60%, and 70%), solid-liquid ratio (1:10, 1:20, 1:30, 1:40, 1:50, 1:60, and 1:70 g/mL), ultrasound temperature (30, 40, 50, 60, 70, 80, and 90 °C), and ultrasound time (10, 20, 30, 40, 50, and 60 min)) were systematically studied.

The extraction rate of crude CYPP was defined as the mass ratio of CYPP to the initial feedstock mass. The total saccharide content of CYPP was determined by the phenol‑sulfuric acid method (DuBois et al., 1956). Briefly, 1 mL of polysaccharide solution (0.05 mg/mL) was mixed with 1 mL of 6% phenol solution and 5 mL of sulfuric acid. The mixture was incubated in a boiling water bath for 15 min, then cooled to room temperature. The absorbance of the mixture was measured at 490 nm, and the total saccharide content was calculated based on d-glucose standard curve. The total saccharide extraction rate (*Y*_t_) of CYPP was calculated by Eq. (2):

(2) $$Y_{t}=\left(Y_{c}\times C_{t}\right)\times 100\%$$

where *Y*_c_ is the crude CYPP extraction rate and *C*_t_ is the total saccharide content.

### 2.4 Deproteinization of CYPP by enzyme method

The proteins are mainly impurities in crude polysaccharides, which may result in an ambiguous interpretation in structural characterization and activity assays of polysaccharides. The protein content in CYPP was determined by Coomassie brilliant blue method (Cisternas-Novoa et al., 2014). The deproteinization rate of crude CYPP was defined as the ratio of AF4-UV area of the enzyme-treated CYPP to that of the non-deproteinized CYPP (Fig. S1). In this study, three proteases (i.e., alkaline proteinase, dispase, and papain) were selected for deproteinization of CYPP. Briefly, 100 mg of crude CYPP and 100 mL of deionized water were added to a 200 mL beaker. The mixture was stirred at 70 °C and 300 rpm for 2 h to obtain CYPP solution with a concentration of 1 mg/mL.

Alkaline proteinase: Adjust the pH of the CYPP solution to 9. Then, 1.00 mL alkaline protease solution (200 U/mL) was mixed with the CYPP solution and stirred at 45 °C and 300 rpm for 0.5 h (Liu et al., 2021). The CYPP solution was heated at 100 °C for 10 min to inactivate the enzyme. Finally, the CYPP solution was cooled to room temperature, and the pH was adjusted to 7. The precipitate was removed via centrifugation at 4000*g* for 10 min.

Dispase: Adjust the pH of the CYPP solution to 7. Then, 0.80 mL dispase solution (250 U/mL) was mixed with the CYPP solution and stirred at 35 °C and 300 rpm for 2.5 h (Zeng et al., 2019). The CYPP solution was heated at 100 °C for 10 min to inactivate the enzyme. The CYPP solution was cooled to room temperature, and the pH was adjusted to 7. The precipitate was removed via centrifugation at 4000*g* for 10 min.

Papain: Adjust the pH of the CYPP solution to 6. Then, 0.25 mL papain solution (800 U/mL) was mixed with the CYPP solution and stirred at 70 °C and 300 rpm for 1.0 h (Ahmad Nadzri et al., 2021). Then, the CYPP solution was heated at 100 °C for 10 min to inactivate the enzyme. The CYPP solution was cooled to room temperature, and the pH was adjusted to 7. The precipitate was removed via centrifugation at 4000*g* for 10 min.

### 2.5 Extraction of CYPP by ultrasound-assisted NADES and water extraction methods

In this study, XCYPPs extracted using ultrasound-assisted NADES and ultrasound-assisted water extraction methods were denoted as XCYPP-N and XCYPP-W, respectively, whereas TCYPPs extracted by both methods were denoted as TCYPP-N and TCYPP-W, respectively. Briefly, the mixture of *Chinese yam* peel powder (10 g) and deionized water or NADES (400 mL) was sonicated in a KQ5200DE ultrasound apparatus (Kunshan Ultrasound Instrument Co., Ltd., Jiangsu, China) at 70 °C for 40 min. The extract was centrifuged at 4000*g* for 10 min and the supernatant was deproteinized by the enzyme method as described in Section 2.4. After removing the denatured proteins by centrifugation, the supernatant was added with 95% ethanol to precipitate CYPP. After centrifugation, the precipitation was lyophilized to obtain four CYPP samples (denoted as XCYPP-N, XCYPP-W, TCYPP-N, and TCYPP-W, respectively). The total saccharide content and uronic acid content of CYPP were determined by the phenol‑sulfuric acid method and *m*-hydroxydiphenyl method, respectively (Buysse & Merckx, 1993; Zhao et al., 2024).

### 2.6 Monosaccharide composition analysis of CYPPs

The monosaccharide compositions of CYPP were analyzed according to previously reported method with minor modifications (Cao et al., 2019). A 7.0 mg CYPP was hydrolyzed into monosaccharide by TFA (3.0 mL, 2 M) in an oil bath at 120 °C for 4 h and then cooled in an ice-water bath for 10 min. The solvent was removed by rotary evaporation at 65 °C. The residue was washed with 1 mL of methanol and evaporated to dryness at 37 °C. This process was repeated three times to remove excess TFA. The residue was then redissolved in 3 mL of water to obtain the polysaccharide solution. The polysaccharide solution (250 μL) was mixed with NaOH (250 μL, 0.6 M) and PMP-methanol (500 μL, 0.4 M), then incubated at 70 °C for 1 h. After cooling to room temperature, the reaction mixtures were neutralized with HCl (500 μL, 0.3 M), and then extracted four times with chloroform. The monosaccharide standard solution (Man, Rib, Rha, GlcA, GalA, Glc, Gal, Xyl, Ara, and Fuc) was derivatized under the same conditions as CYPP. The aqueous supernatant was filtered through a 0.22 μm membrane filter prior to monosaccharide composition analysis by a 1200 high performance liquid chromatography (HPLC) (Agilent Technologies, Waldbronn, Germany) with a BioReags AQ-C18 column (4.6 mm × 250 mm 5 μm 200 Å). The mobile phase consisted of acetonitrile and monopotassium phosphate-NaOH buffer solution (pH 6.7, 0.05 M) at the ratio of 16:84 (v/v). Injection volume was 10 μL. The flow rate was set at 1.0 mL/min and the column temperature was maintained at 35 °C.

### 2.7 Asymmetrical flow field-flow fractionation (AF4) analysis of CYPP

The Eclipse AF4 system (Wyatt Technology, Dernbach, Germany) was employed to characterize the structure and conformation of CYPP. The AF4 system was connected to an SPD-20A UV detector (Shimadzu Corporation, Kyoto, Japan) at a wavelength of 280 nm, a DAWN HELEOS II MALS detector (Wyatt technology, Santa Barbara, CA, USA) with a calibration constant of 3.483 × 10^−5^, and a RID-20A dRI detector (Shimadzu Corporation, Kyoto, Japan). AF4-MALS-dRI can provide the structural and conformational information including (*M*_w_, *R*_g_, *ρ*_app_, and shape factor) (Guo et al., 2019). The Wyatt long channel was assembled with a 350 μm-thickness Mylar spacer and a regenerated cellulose ultrafiltration membrane with a molecular weight cutoff of 10 kDa. Deionized water containing 5 mM NaNO_2_ and pH 7.0 was used as the carrier liquid. The carrier liquid was filtered through a 0.22 μm regenerated cellulose membrane filter prior to use. One hundred μL sample with a concentration of 1 mg/mL was injected into the channel at the flow rate of 0.2 mL/min for 0.9 min. The detector flow rate was kept constant at 1.0 mL/min. The crossflow rate and half-life were optimized and the results were shown in Fig. S2. The optimized crossflow rate started at 2.0 mL/min and decreased exponentially to 0.1 mL/min with a half-life of 1.8 min. The performance of AF4 was validated by running BSA prior to CYPP analysis.

### 2.8 Fourier transform infrared spectroscopy (FTIR) analysis

Two mg CYPP was mixed with 200 mg KBr. The mixture was placed in the mold and pressed into transparent sheet with a 10 MPa hydraulic machine. Fourier transform infrared spectroscopy (FTIR) characterization of the sample was performed according to a previously reported method with slight modifications (Liu et al., 2023). The FTIR spectra of CYPP samples were collected using a Nicolet iS50 FTIR spectrometer (Thermo Nicolet Inc., Waltham, USA) with 32 scans at a resolution of 4 cm^−1^ in the wavenumber range of 4000–500 cm^−1^. Background spectra were acquired in air and automatically subtracted by the OPUS software (Version 8.5, Bruker Optics, Ettlingen, Germany).

### 2.9 Congo red assay

Within an appropriate concentration range, NaOH can induce Congo red to bind with the triple-helix structure of polysaccharides, forming a complex, which exhibits a red shift in maximum absorption wavelengths (λ_max_) (Zhang et al., 2021). Thus, Congo red assay is commonly used to estimate the triple-helix structure of polysaccharides. One mL of CYPP solution (1 mg/mL) and 1 mL of Congo red solution (0.056 mg/mL) were added into a centrifuge tube and mixed thoroughly. The NaOH concentration of the solution was adjusted to 0.1, 0.2, 0.3, 0.4, and 0.5 mol/L by adding 1 mol/L NaOH solution. The solution was thoroughly mixed and reacted at room temperature for 10 min. Deionized water was used as a blank group. The maximum absorption wavelengths (λ_max_) of the CYPP samples with different NaOH concentrations were measured in the wavelength range of 400–600 nm using a Cary 60 UV–Visible spectrophotometer (Agilent Technology Inc., San Jose, CA, USA).

### 2.10 In vitro chemical antioxidant activity of CYPP

#### 2.10.1 DPPH free radical scavenging capacity

The DPPH free radical scavenging capacity was determined by the method of Deng et al. with slight modifications (Deng & Huang, 2025). Briefly, 2.0 mL of CYPP solution with different concentrations (1, 2, 3, 4, 5, and 6 mg/mL) was mixed with the same volume of DPPH-ethanol solution (0.5 mmol/L). The reaction was carried out in the dark for 30 min, and the absorbance values of the solutions were measured at 517 nm. Absolute ethanol replaced the sample and DPPH solution as the blank group and the control group, respectively. Vitamin C (Vc) was used as the positive group. DPPH free radical scavenging capacity of CYPP was calculated by Eq. (3):

(3) $$\left[1-\frac{\left(A_{1}-A_{2}\right)}{A_{0}}\right]\times 100\%$$

where *A*_1_ is the absorbance of the sample group, *A*_2_ is the absorbance of the control group, and *A*_0_ is the absorbance of the blank group.

#### 2.10.2 ABTS^+^ free radical scavenging capacity

ABTS^+^ free radical scavenging capacity assay kit was used to evaluate the ABTS^+^ free radical scavenging capacity of CYPP according to the manufacturer's instructions (BC4770, Solarbio Life Sciences, Beijing, China). Briefly, 50 μL of CYPP solutions at different concentrations (1, 2, 3, 4, 5, and 6 mg/mL) were mixed with 950 μL of ABTS^+^ working solution, and allowed to stand at room temperature in the dark for 6 min. The CYPP solution was used as the sample group, and Vc was used as the positive group. ABTS^+^ free radical scavenging capacity of CYPP was determined by the same method used for DPPH free radical, except that the absorbance values of the solutions were measured at 405 nm.

#### 2.10.3 Hydroxyl free radical scavenging capacity

Hydroxyl free radical scavenging capacity assay kit was used to evaluate the hydroxyl free radical scavenging capacity of CYPP according to the manufacturer's instructions (BC1325, Solarbio Life Sciences, Beijing, China). Briefly, 50 μL of CYPP solutions at different concentrations (1, 2, 3, 4, 5, and 6 mg/mL) were mixed with 300 μL of the hydroxyl radical working solution, and the mixture was incubated in a constant temperature water bath at 37 °C in the dark for 30 min. After cooling to room temperature, the mixture was centrifuged at 11,000*g* for 10 min. The CYPP solution was used as the sample group, and Vc was used as the positive group. Hydroxyl free radical scavenging capacity of CYPP was determined by the same method used for DPPH free radical, except that the absorbance values of the solutions were measured at 536 nm.

#### 2.10.4 Total antioxidant capacity

Total antioxidant capacity (T-AOC) assay kit was used to evaluate the T-AOC of CYPP according to the manufacturer's instructions (BC1315, Solarbio Life Sciences, China). Briefly, 30 μL of CYPP solutions at different concentrations (1, 2, 3, 4, 5, and 6 mg/mL) were mixed with 990 μL of T-AOC working solution, and allowed to stand at room temperature for 10 min. The CYPP solution was used as the sample group, and Vc was used as the positive group. T-AOC of CYPP was determined by the same method used for DPPH free radical, except that the absorbance values of the solutions were measured at 593 nm.

### 2.11 Antioxidant protection of CYPP for H_2_O_2_-induced HepG2 cells

MTT assay for cell viability was performed according to the method described by Chung et al. (2015). The MTT cell proliferation and cytotoxicity assay kit was used to determine the cytotoxicity of CYPP according to the manufacturer's instructions (M1020, Solarbio Life Sciences, Beijing, China). HepG2 cells were seeded in 96-well plates at a density of 5 × 10^4^ cells/well. The cells were incubated in DMEM supplemented with 10% FBS at 37 °C for 24 h in a 5% CO_2_ atmosphere. To investigate the protective effect of CYPP on oxidative damage, HepG2 cells were treated with H_2_O_2_ (500 μM) for 12 h, and then the CYPP of different concentrations (50, 100, 200, 400, and 800 μg/mL) was added to the culture medium and incubated at 37 °C for 24 h. After incubation, 10 μL MTT was added to each well and incubated for 30 min, the supernatant was then removed and 150 μL DMSO was added to solubilize the formazan. Absorbance at 490 nm was determined using a microplate reader (Infinite 200 PRO, Männedorf, Switzerland).

### 2.12 Data treatment

The AF4-MALS-dRI data were processed by using the Astra software (Version 8.1.1, Wyatt Technology, Santa Barbara, CA, USA). The *M*_w_ and *R*_g_ distributions of CYPP were determined based on the Berry method (Andersson et al., 2003) with a refractive index increment (*d*_n_/*d*_c_) of 0.146 mL/g (Belloperez et al., 1998). The *ρ*_app_ of CYPP was calculated using MatLab 2023 (MathWorks, Natick, MA, USA) based on its *M*_w_ and *R*_g_. All experiments were conducted in triplicate, unless stated otherwise. The results are expressed as mean ± standard deviation (SD). The error bars shown in figures represent SD. The data were analyzed by one-way analysis of variance (ANOVA), followed by Duncan's multiple range test by using IBM SPSS 23 Statistical Software Program. The differences were considered statistically significant at *p* < 0.05. Correlation analysis was carried out by the Pearson correlation coefficient in Origin 2021 software.

## 3 Results and discussion

### 3.1 Effect of the type of NADES on the extraction rate of CYPP

**Fig. 1:**
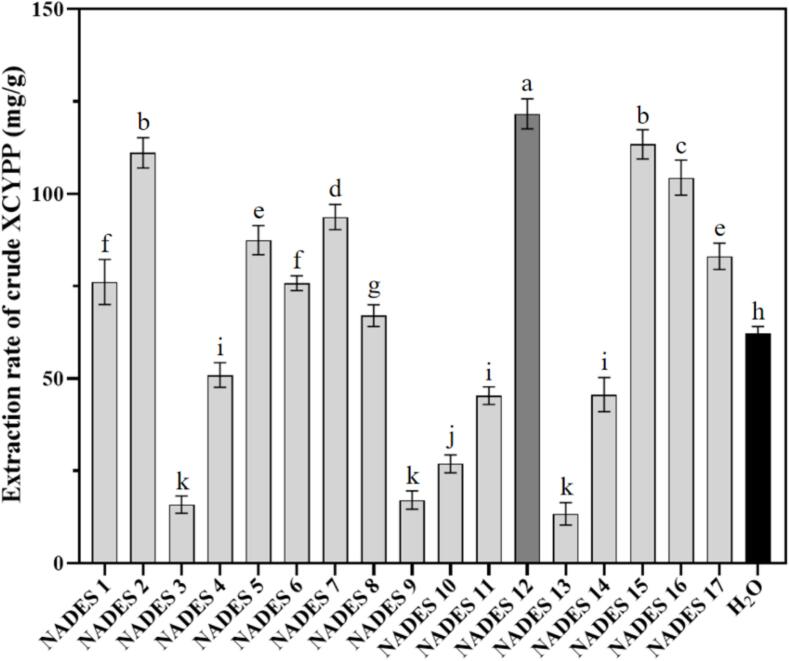
Effect of the type of natural deep eutectic solvents (NADES) on the extraction rate of crude *Xiaobaizui Chinese yam* polysaccharide (XCYPP).

The physical properties of NADESs (such as viscosity, pH, and polarity) are related to the compositions of HBA and HBD, and affect the extraction rate of polysaccharides (Md Yusoff & Shafie, 2025). In this study, three types of HBA (i.e., ChCl, BET, and Pro) were combined with seven types of HBD (carboxylic acids, alcohols, sugar, and amides) to prepare 17 types of NADES, as shown in Table 1. The viscosity of NADES composed of amide (Urea) HBD was the lowest regardless of the type of HBA investigated in this study. The pH value of NADES was primarily dependent on the type of HBD: NADES containing carboxylic acids (i.e., CA and MA) HBD displayed smaller pH values, followed by sugar HBD (Fru) and alcohols HBD (i.e., PG and Xyl). Carboxylic acids HBD (i.e., CA and MA) led to a low pH value of NADES, which may degrade polysaccharides, thereby affect their bioactivities (Al-Akayleh et al., 2025). The amide HBD (i.e., Urea) exhibited the largest pH value. *Xiaobaizui Chinese yam* peel was selected for studying the effect of NADES extraction factors on the extraction rate of XCYPP. The effect of the type of NADES on the extraction rate of crude XCYPP is illustrated in Fig. 1. The extraction rate of XCYPP-N varied significantly among the 17 types of NADES. Ten types of NADES had higher extraction rates compared with water extraction method. The enhancement of the extraction rate of XCYPP-N was mainly attributed to the robust hydrogen bond network formed within NADES, which could efficiently disrupt the cellulose and pectin structure in *Chinese yam* peel cell walls to release encapsulated polysaccharides, and form strong interactions with hydroxyl and carboxyl groups of polysaccharides, thereby improving the extraction rate of XCYPP (Md Yusoff et al., 2025). Moreover, the viscosity, pH, and polarity of NADES might affect the extraction rate of XCYPP-N (Feng et al., 2023). The NADES 12 composed of BET and Urea achieved the highest extraction rate of XCYPP-N (121.63 ± 3.35 mg/g), which was 1.96-fold higher than that of XCYPP-W (62.13 ± 1.56 mg/g). The NADES 13 composed of BET and Gly exhibited the lowest extraction rate of XCYPP-N (13.37 ± 2.48 mg/g). This may be due to the highest viscosity (36.59 ± 0.64 mm^2^/s) of NADES 13, which compromised NADES fluidity and consequently limited interaction with XCYPP (Yücel et al., 2025).

**Fig. 2:**
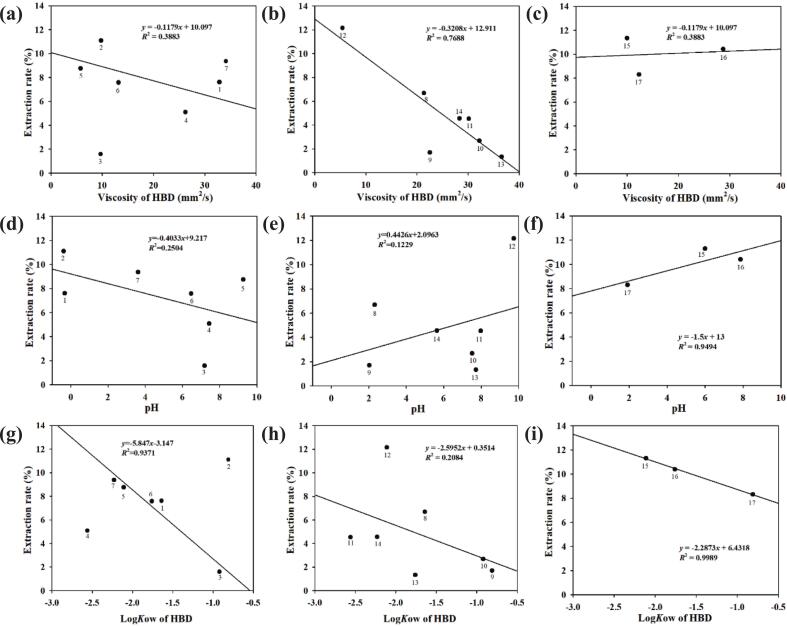
Relationship between viscosity, pH, and polarity of NADES and the extraction rate of XCYPP-N. (a, d, and g): ChCl-HBD groups; (b, e, and h): BET-HBD groups; (c, f, and i): Pro-HBD groups. (Log*Kow* value from the National Biotechnology Information Center).

The relationships between viscosity, pH, and polarity of NADES and the extraction rate of XCYPP-N are illustrated in Fig. 2. The viscosity of NADES significantly affected the extraction rate of XCYPP-N. NADES with low viscosity enhanced the mobility of *Chinese yam* peel powder within the solvent and promoted the dissolution of XCYPP, thereby improving the extraction rate of XCYPP-N. Conversely, NADES with high viscosity reduced solvent mobility, decreased contact between NADES and XCYPP, resulting in low extraction rate of XCYPP-N. Overall, the extraction rate of XCYPP-N extracted by ChCl-HBD group and BET-HBD group gradually decreased with increasing viscosity of NADES. It should be noted that the extraction rate of XCYPP-N extracted by NADES 3 slightly deviated from the trend (Fig. 2(a)), and the viscosity of Pro-HBD NADES groups exhibited no significant impact on the extraction rate of XCYPP-N (Fig. 2(c)). The results suggested that the viscosity of NADES was not the sole factor affecting the extraction rate of XCYPP-N.

The pH of NADES is primarily dependent on the type of HBD and affects the extraction rate through proton-donating and electron-accepting capacities. Under acidic or alkaline conditions, NADESs could weaken electrostatic repulsion between polysaccharides molecules, thereby enhancing the solubility and extraction rate of XCYPP-N (Dai et al., 2025). Generally, the extraction rate of ChCl-HBD group gradually decreased with increasing pH, while the BET-HBD and Pro-HBD groups exhibited an opposite trend. This discrepancy may arise from ChCl's instability under alkaline conditions, whereas in acidic environment, NADES had more active functional groups that facilitated the formation of robust hydrogen bond network with polysaccharides, thereby promoting the extraction rate of XCYPP-N (Zhang, Lin, et al., 2024). In contrast, under alkaline conditions, the functional groups (such as carboxyl groups) of BET and Pro may undergo deprotonation, enhancing their ability as HBA and forming a more effective hydrogen bond network with polysaccharides, thereby enhancing the extraction rate of XCYPP-N.

Log*Kow* (logarithm of the *n*-octanol/water partition coefficient), a hydrophobicity index, reflects the polarity of NADES (Zhang, Li, et al., 2024). Higher Log*Kow* value (lower polarity) indicates stronger hydrophobicity. For NADES composed of the same HBA groups, the hydrophobicity of NADES was correlated closely with the Log*Kow* of HBD. NADES with Log*Kow* < 10 is classified as hydrophilic solvent. The results (Fig. 2(g–i)) showed that higher Log*Kow* values of NADESs resulted in a lower XCYPP-N extraction rate. This can be explained by “like-dissolves-like” principle, the polar XCYPP molecules were more readily extracted by NADES with higher polarity. The results suggested that NADES with lower viscosity, higher pH value, and larger polarity was conducive to the enhancement of the extraction rate of XCYPP-N. Thus, the NADES composed of BET and Urea was selected as the extraction solvent for the subsequent experiments.

**Fig. 3:**
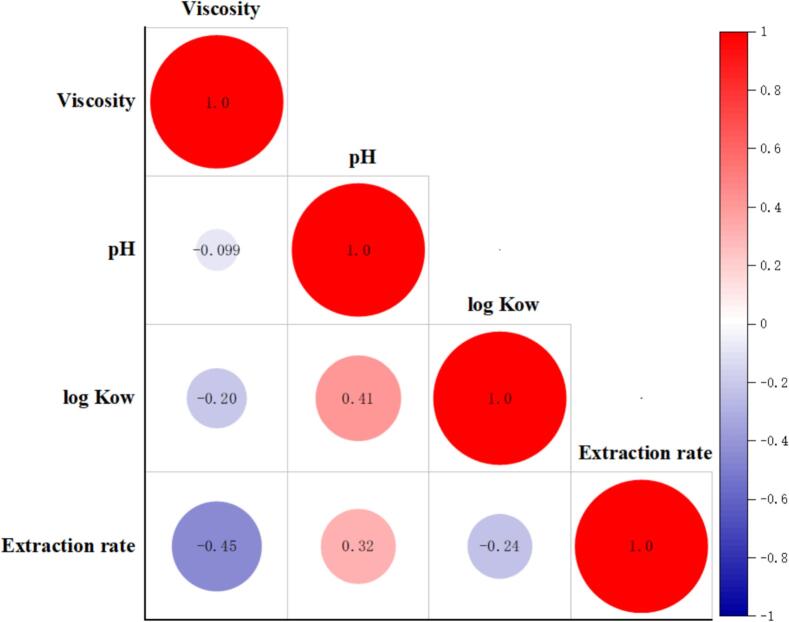
Correlation analysis of viscosity, pH, and polarity of NADES with the extraction rate of XCYPP-N.

To investigate the correlation between the physical properties of NADES and the extraction rate of XCYPP-N, the viscosity, pH, and polarity of NADES were analyzed against the extraction rate of XCYPP-N using Pearson correlation coefficient. A correlation heatmap of the physical properties of NADES and the extraction rate of XCYPP-N is displayed in Fig. 3. The red indicates positive correlation, blue represents negative correlation, and color intensity reflects the extent of the correlation. The result revealed that the viscosity of NADES exhibited a moderate negative correlation with the extraction rate of XCYPP-N, with an absolute correlation coefficient (|r|) of 0.45. The pH of NADES showed a weak positive correlation (|r| = 0.32), while the polarity of NADES showed a weak negative correlation (|r| = 0.24). The result indicated that the viscosity of NADES was the primary factor affecting the extraction rate of XCYPP-N.

### 3.2 Effect of extraction conditions on the extraction rate and total saccharide content of XCYPP-N

#### 3.2.1 Molar ratio of NADES

**Fig. 4:**
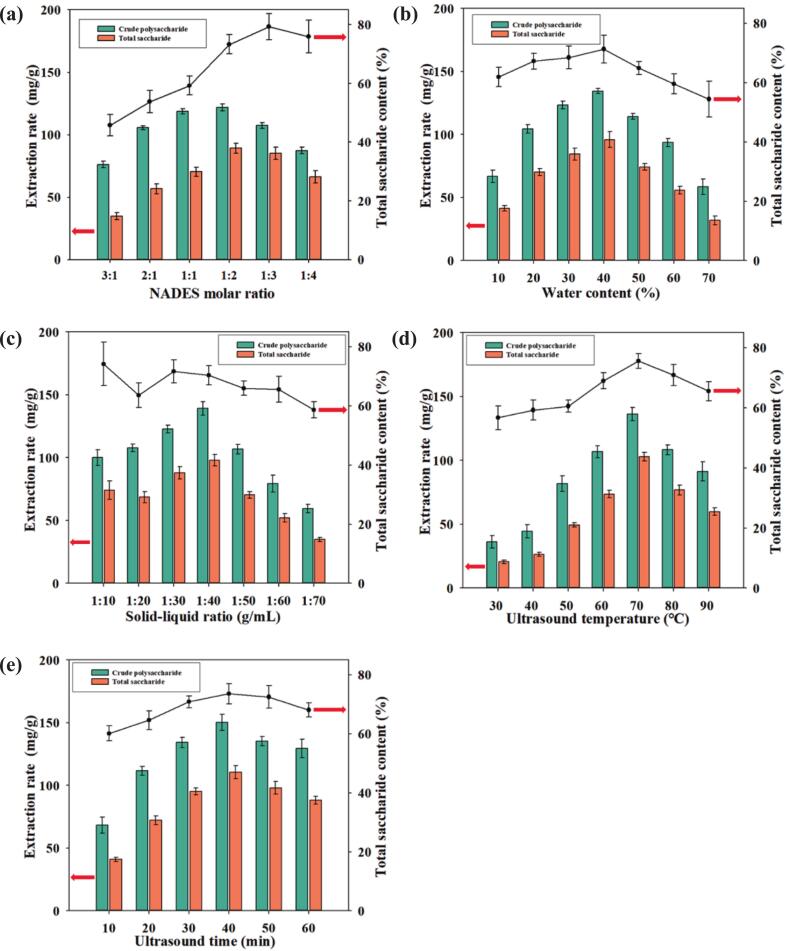
Effect of extraction conditions on the extraction rate and total saccharide content of XCYPP-N. (a): NADES molar ratio; (b): water content; (c): solid-liquid ratio; (d): ultrasound temperature; (e): ultrasound time.

The molar ratio of HBA to HBD in NADES governs the strength of hydrogen bond interactions within the solvent, thereby affecting the solubility and extraction rate of polysaccharides (Rahman et al., 2025). The effect of molar ratio of HBA to HBD on the extraction rate of XCYPP-N is shown in Fig. 4(a). The other extraction conditions for XCYPP-N were as follows: water content was 30%, solid-liquid ratio was 1:30 g/mL, ultrasound temperature was 70 °C, and ultrasound time was 30 min. When the molar ratio of BET to Urea was adjusted from 3:1 to 1:4, the extraction rate and total saccharide content of XCYPP-N exhibited a similar trend of initially increase followed by a decrease. When the molar ratio of BET to Urea increased, the carboxyl groups of BET could form more hydrogen bond networks with the amino and carbonyl groups of Urea, promoting the dissolution of XCYPP and thereby increasing the extraction rate and total saccharide content of XCYPP-N. At the molar ratio of 1:2, the XCYPP-N extraction rate achieved maximum value of 121.83 ± 2.86 mg/g. When the molar ratio of BET to Urea further increased to 1:3 and 1:4, the XCYPP-N extraction rate decreased. This can be explained by the fact that more Urea content not only can enhance hydrogen bond interactions, but also elevate the viscosity of NADES, which is not beneficial for dissolving XCYPP-N (Qu et al., 2023). Notably, the maximum total saccharide content of XCYPP was obtained at the molar ratio of 1:3. One possible explanation is that the increased Urea content preferentially engaged in the formation of hydrogen bonds with hydroxyl groups of XCYPP, enhancing polysaccharide solubility, thereby elevating total saccharide content of XCYPP-N. However, when the molar ratio of BET to Urea increased to 1:4, the elevated viscosity of the NADES restricted polysaccharide mobility, resulting in a decrease of the total saccharide content. Thus, the molar ratio of BET to Urea of 1:2 was selected for the subsequent experiments.

#### 3.2.2 Water content of NADES

The viscosity of NADES can be modulated by adjusting its water content (Koh et al., 2023). As shown in Fig. 4(b), the extraction rate and total saccharide content of XCYPP-N gradually increased with increasing water content and achieved maximum values of 134.53 ± 1.96 mg/g and 71.34 ± 4.69%, respectively, at the water content of 40%. After that, the extraction rate and total saccharide content of XCYPP-N decreased with increasing water content. The viscosity of NADES reduced with more water, which enhanced the mobility of XCYPP in NADES, thereby improving the extraction rate and total saccharide content of XCYPP-N. However, when water content exceeded 40%, the extraction rate and total saccharide content of XCYPP-N gradually declined. This may be attributed to the fact that the excessive water disrupted the hydrogen bond network of NADES, thereby weakening interactions between XCYPP and NADES, resulting in a decrease in the extraction rate and total saccharide content of XCYPP-N (Yang, Li, & Li, 2025). The water content of 40% was selected for the subsequent experiments.

#### 3.2.3 Solid-liquid ratio

As illustrated in Fig. 4(c), the extraction rate and total saccharide content of XCYPP-N gradually increased with increasing solid-liquid ratio in the range of 1:10 to 1:40 g/mL. This can be explained by the fact that the concentration and viscosity of the extraction system decreased as the liquid increased. During the subsequent XCYPP-N extraction process, the enhanced ultrasonic cavitation effect promoted the disruption of *Chinese yam peel* cell walls, allowing XCYPP to be released easily (Tang et al., 2023). However, as the solid-liquid ratio further increased from 1:40 to 1:70 g/mL, both extraction rate and total saccharide content gradually decreased, which may be attributed to excessive liquid volume, reducing the ultrasound energy density per unit volume, thereby weakening the interactions between XCYPP and NADES and increasing the impurity dissolution during the extraction process (Guan et al., 2025). Considering the extraction rate and total saccharide content of XCYPP-N, the solid-liquid ratio of 1:40 g/mL was selected for the subsequent experiments.

#### 3.2.4 Ultrasound temperature

Fig. 4(d) shows the effect of ultrasound temperature on the extraction rate and total saccharide content of XCYPP-N. When the ultrasound temperature increased from 30 °C to 70 °C, the extraction rate and total saccharide content of XCYPP-N gradually increased and achieved maximum values of 136.17 ± 5.19 mg/g and 75.59 ± 2.47%, respectively. This may be attributed to the fact that the high ultrasound temperature enhanced molecular thermal motion, which promoted the dissolution of XCYPP from *Chinese yam* peel powder into the NADES (Han, et al., 2025). Additionally, the high ultrasound temperature reduced the viscosity of NADES, thereby accelerating the mass transfer and diffusion rate of XCYPP within the NADES, consequently enhancing the extraction rate and total saccharide content of XCYPP-N. However, when the ultrasound temperature further increased from 70 °C to 90 °C, both extraction rate and total saccharide content of XCYPP-N decreased, which may be due to the degradation of polysaccharides under high temperature conditions (Zhang, Lin, et al., 2024). Thus, ultrasound temperature of 70 °C was selected for the subsequent experiments.

#### 3.2.5 Ultrasound time

The effect of ultrasound time on the extraction rate and total saccharide content of XCYPP-N is shown in Fig. 4(e). Ultrasound time directly affected the contact time between XCYPP and NADES, thereby affecting the extraction rate and total saccharide content of XCYPP-N. When the ultrasound time increased from 10 to 40 min, both the extraction rate and total saccharide content of XCYPP-N gradually increased and achieved maximum values of 150.30 ± 6.45 mg/g (2.42-fold higher than that of XCYPP-W) and 76.73 ± 3.53%, respectively. This phenomenon was attributed to the gradual dissolution of *Chinese yam* peel cell walls under prolonged ultrasound time, which enhanced the solubility of XCYPP through the interaction with NADES. However, further extension of ultrasound time beyond 40 min resulted in a decrease in the extraction rate and total saccharide content of XCYPP-N, which may be due to the degradation of XCYPP (Lai et al., 2025). Thus, 40 min was selected as the optimal ultrasound time.

### 3.3 Effect of the type of enzymes on the deproteinization of CYPP

Proteins were identified as the primary impurity in crude polysaccharides. In this study, an enzyme method was employed for the deproteinization of crude CYPP, and the results are summarized in Table 2. The results revealed that all enzymatic treatments reduced the protein content in the crude XCYPP-N. Alkaline proteinase exhibited the highest deproteinization rate (71.87 ± 2.88%), the smallest residual protein content of 3.97 ± 0.51%, and the highest total saccharide content of 92.89 ± 3.24%. Thus, alkaline proteinase was selected for the subsequent deproteinization of the other CYPP samples.

**Table 2:** Efficiency of deproteinization with different types of enzymes.

Type of enzyme	Protein content^1^ (%)	Deproteinization rate^2^ (%)	Total saccharide content^3^ (%)
Non-deproteinized	20.10 ± 0.87^a^	–	76.73 ± 3.53^b^
Papain	17.91 ± 0.40^b^	4.10 ± 2.74^c^	80.81 ± 3.51^b^
Dispase	16.04 ± 0.41^c^	21.33 ± 3.31^b^	82.18 ± 2.65^b^
Alkaline proteinase	3.97 ± 0.51^d^	71.87 ± 2.88^a^	92.89 ± 3.24^a^

Note: ^1^Determined by Coomassie brilliant blue method; ^2^Calculated based on AF4-UV peak area ratio; ^3^Determined by phenol‑sulfuric acid method. The data are expressed as mean ± standard deviation (*n* = 3). Different superscript letters in the same column represent statistically significant differences (*p* < 0.05).

### 3.4 The total saccharide content, protein content, uronic acid content, and monosaccharide composition analysis of CYPP

In this study, the ultrasound-assisted NADES and ultrasound-assisted water extraction methods were employed to extract polysaccharides from *Xiaobaizui Chinese yam* peel (XCYPP-N, XCYPP-W) and *Tiegun Chinese yam* peel (TCYPP-N, TCYPP-W), followed by alkaline proteinase deproteinization. The total saccharide, protein, and uronic acid contents of CYPP samples are summarized in Table 3. The results demonstrated that NADES extracted CYPP (i.e., XCYPP-N and TCYPP-N) exhibited higher total saccharide contents (92.89 ± 3.24% and 88.43 ± 1.36%, respectively) and significantly lower protein contents (2.39 ± 1.03% and 4.27 ± 1.15%, respectively) compared with those obtained by the ultrasound-assisted water extraction method. This may be due to the fact that the protein surface possesses polar groups (i.e., amino and carboxyl groups), which were more readily adsorbed by the viscous network of NADES and subsequently removed along with the solvent during centrifugation step, thereby enhancing the total saccharide content of CYPP-N (Deng et al., 2025).

**Table 3:** The total saccharide content, protein content, and uronic acid content of CYPP.

Sample	Total saccharide content^1^ (%)	Protein content^2^ (%)	Uronic acid content^3^ (%)
XCYPP-N	92.89 ± 3.24^a^	2.39 ± 1.03^c^	8.39 ± 0.59^a^
XCYPP-W	84.41 ± 0.75^c^	12.23 ± 0.90^b^	7.28 ± 0.24^b^
TCYPP-N	88.43 ± 1.36^b^	4.27 ± 1.15^c^	6.14 ± 0.28^c^
TCYPP-W	79.97 ± 0.85^d^	18.38 ± 0.48^a^	5.31 ± 0.53^c^

Note: ^1^Determined by phenol‑sulfuric acid method; ^2^Determined by Coomassie brilliant blue method; ^3^Determined by *m*-hydroxybiphenyl method. The data are expressed as mean ± standard deviation (*n* = 3). Different superscript letters in the same column represent statistically significant differences (*p* < 0.05).

Polysaccharides are classified as neutral (< 5%) and acidic (≥ 5%) polysaccharides based on uronic acid content. The results showed that the uronic acid contents of four CYPP samples were larger than 5%, demonstrating that all CYPP samples were acidic polysaccharides. Uronic acid content of XCYPP-N was the highest (8.39 ± 0.59%), followed by XCYPP-W (7.28 ± 0.24%), TCYPP-N (6.14 ± 0.28%), and TCYPP-W (5.31 ± 0.53%). The results indicated that extraction method and *Chinese yam* variety affected the uronic acid content of CYPP. Notably, the uronic acid content of XCYPP was higher than that of TCYPP regardless of extraction method. For the CYPP from the same variety, the uronic acid content of the CYPP samples extracted by NADES was higher than that of the water-extracted CYPP samples. When BET-Urea was used as CYPP extraction medium, the interactions (including electrostatic forces, hydrogen bond, and van der Waals forces) between NADES and CYPP were stronger than those in water. Especially, the positively charged regions of BET-Urea can strongly interact with the negatively charged parts in polysaccharide monomers (and vice versa), which could facilitate the dissolution of acidic polysaccharides containing uronic acids, thereby leading to a high uronic acid content (Md Yusoff & Shafie, 2025).

The PMP-HPLC results (Fig. S3) revealed that the four extracted CYPPs were composed of three main monosaccharides (i.e., Man, Glc, and Gal). The molar ratios of Man, Glc, and Gal in XCYPP-N, XCYPP-W, TCYPP-N, and TCYPP-W were 2.05:69.83:28.04, 11.73:62.22:26.08, 0.74:70.92:28.32, and 3.48:69.21:27.30, respecti*v*ely. The Glc content in CYPPs extracted by NADES was higher than those obtained using the hot water extraction method.

### 3.5 AF4 analysis of CYPP

**Fig. 5:**
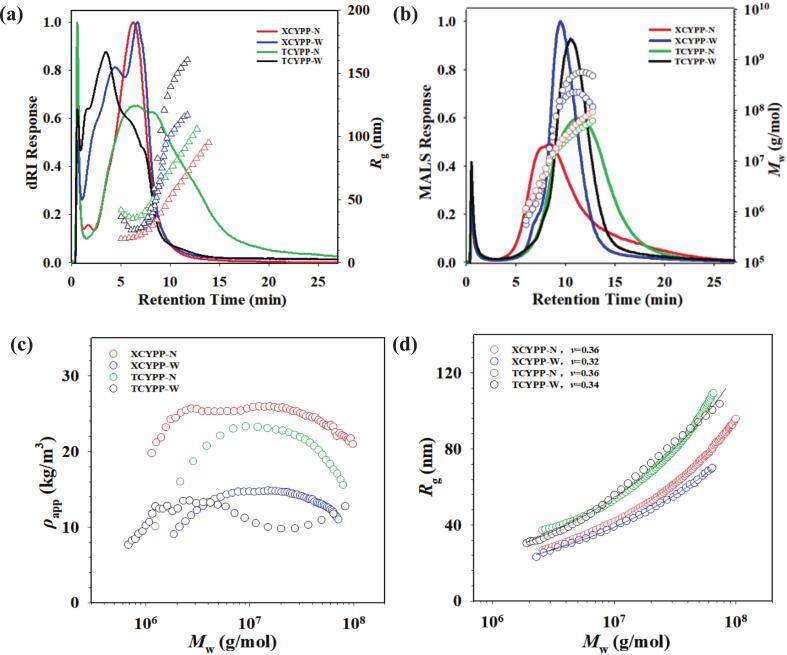
AF4-MALS-dRI and conformation fractograms, *R*_g_, *M*_w_, and *ρ*_app_ distributions of CYPP samples. (a): AF4-dRI fractogram and *R*_g_ distributions; (b): AF4-MALS fractogram and *M*_w_ distributions; (c) *ρ*_app_ distributions of CYPP; (d) conformation of CYPP.

The performance of AF4-MALS-dRI was *v*alidated by running a standard sample (BSA) prior to sample analysis. The result demonstrated that AF4 had a good performance (Fig. S4). The separation and characterization results of the four CYPP samples by AF4-MALS-dRI are illustrated in Fig. 5. The sample recovery was 88.5%, suggesting that part of the sample may adsorb on the surface of membrane and/or pass through the membrane. The results shown in Fig. 5(a) and (b) indicated that CYPPs from the different varieties exhibited different *M*_w_ and *R*_g_ distributions (listed in Table 4). The extraction method also affected the *M*_w_ and *R*_g_ distributions of CYPP. It can be seen from Fig. 5(a) that the AF4-dRI peak of TCYPP-N exhibited a broader size distribution than that of TCYPP-W. The result indicated that the population with larger size in TCYPP could be extracted by NADES. One eluting peak approximately at 3 min was observed in Fig. 5(a) for the water extracted CYPP, which mainly corresponded to the residual proteins as demonstrated by AF4-UV fractograms (Fig. S5).

**Table 4:** The physical properties of CYPP.

Sample	*M*_w_ distribution (g/mol)	$\bar{M_{w}}$(g/mol)	*R*_g_ distribution (nm)	$\bar{R_{g}}$ (nm)	*ρ*_app_ (kg/m^3^)
XCYPP-N	3.79 × 10^5^–1.18 × 10^8^	4.95 × 10^6^ ± 0.04^a^	18.81–90.05	35.1 ± 0.7^a^	21.16
XCYPP-W	5.56 × 10^5^–2.29 × 10^8^	22.8 × 10^6^ ± 0.3^c^	32.66–115.71	68.9 ± 0.8^c^	12.23
TCYPP-N	2.57 × 10^6^–9.96 × 10^7^	20.1 × 10^6^ ± 0.4^b^	35.09–105.88	58.9 ± 0.6^b^	18.27
TCYPP-W	6.66 × 10^5^–5.68 × 10^8^	51.8 × 10^6^ ± 2.0^d^	25.67–160.62	94.0 ± 1.1^d^	11.53

Note: *M*_w_: Weight average molecular weight, $\bar{M_{w}}$: Average weight average molecular weight, *R*_g_: Radius of gyration, $\bar{R_{g}}$: Average radius of gyration, *ρ*_app_: Apparent density. The data are expressed as mean ± standard deviation (*n* = 3). Different superscript letters in the same column represent statistically significant differences (*p* < 0.05).

Fig. 5(c) shows the *ρ*_app_ of four CYPP samples. The *ρ*_app_ values of XCYPP-N, TCYPP-N, XCYPP-W, and TCYPP-W were 21.16, 18.27, 12.23, and 11.53 kg/m^3^, respectively (Table 4). The results revealed that the *ρ*_app_ of CYPP-N was significantly larger than that of CYPP-W, indicating that the NADES extracted CYPP had a relatively compact structure. This can be interpreted by the fact that ultrasound-assisted NADES extraction had a strong affinity for the polar groups of CYPP, which enhanced the intra−/intermolecular interactions, and further compressed the spacing between the polysaccharide molecular chains, thus resulting in a more compact structure (Subhash et al., 2024).

The conformational information of CYPP was investigated based on the slope of the logarithmic curves of log*R*_g_ versus *v* log*M*_w_. The *v* is the scaling exponent, which is commonly used to evaluate the conformation of samples (Kang et al., 2021). The *v* of 1 represents a rod-like conformation, *v* of 0.5–0.6 indicates a random coil conformation, and *v* of 0.28–0.35 represents a highly branched conformation (Hoyos-Leyva et al., 2017). As shown in Fig. 5(d), the *v* values of XCYPP-N, XCYPP-W, TCYPP-N, and TCYPP-W were 0.36, 0.32, 0.36, and 0.34 respectively, indicating that all CYPP samples had a highly branched conformation.

### 3.6 FTIR analysis of CYPP

**Fig. 6:**
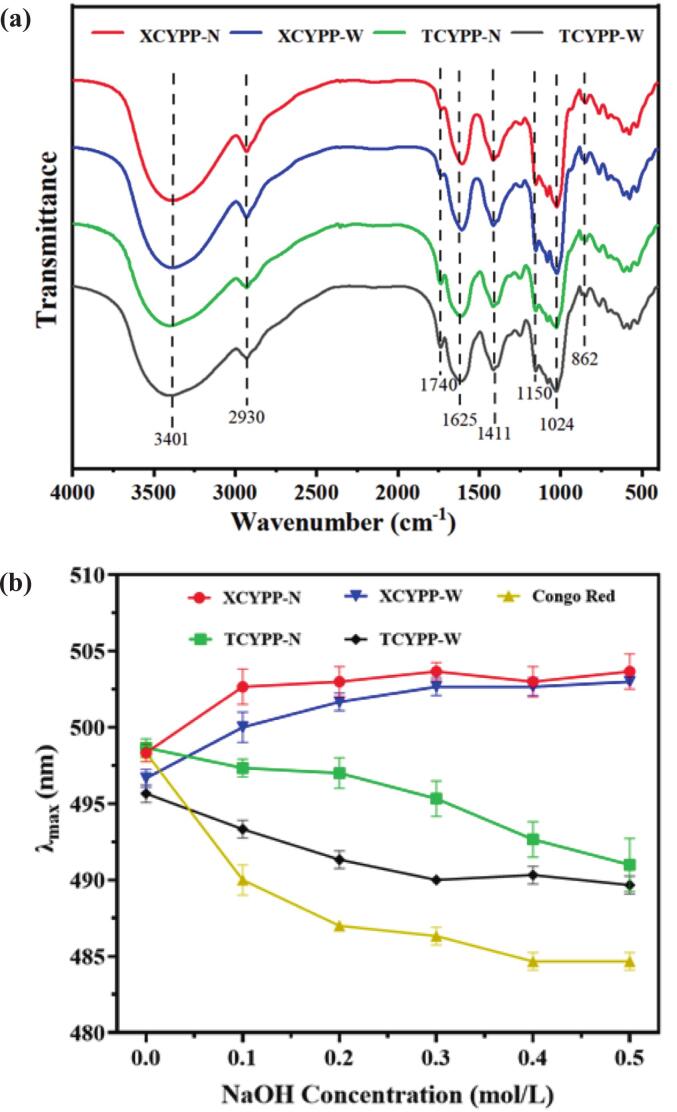
FTIR spectra of CYPP (a) and trends of λ_max_ of CYPP and Congo red complex with different NaOH concentrations (b). (For interpretation of the references to color in this figure legend, the reader is referred to the web version of this article.)

The structure of CYPP was qualitatively analyzed by FTIR. Fig. 6(a) displays the FTIR spectra of four CYPP samples. The strong absorption peak at 3401 cm^−1^ corresponded to O—H stretching vibration, while the peak at 2930 cm^−1^ arose from C—H stretching and bending vibrations. The absorption peaks at 1740 cm^−1^ and 1625 cm^−1^ were attributed to the asymmetric and symmetric stretching vibrations of C=O bonds, indicating the existence of uronic acids (Yan et al., 2025). The peak at 1024 cm^−1^ was primarily determined by C—O—C glycosidic bond vibrations in pyranose ring (Ma et al., 2024). All CYPP samples exhibited weak absorption peaks at 862 cm^−1^, suggesting the presence of *β*-configuration pyranose sugars. No significant difference in functional groups was observed for four CYPP samples, suggesting that the extraction methods did not alter functional groups of CYPP.

### 3.7 Congo red assay of CYPP

It was reported that the polysaccharides with a triple-helix structure exhibited superior bioactivity compared with those without triple-helix structure (Peng et al., 2025). The Congo red assay is widely used to determine the triple-helix structure of polysaccharides. Polysaccharides with a triple-helix structure can form complexes with Congo red in NaOH solution, resulting in a red shift of the maximum absorption wavelength (λ_max_). The results suggested that XCYPP regardless of extraction method had a triple-helix structure as indicated by a significant red shift in λ_max_ (Fig. 6(b)). No red shift of λ_max_ for TCYPP samples was observed, suggesting the absence of triple-helix structure in TCYPP. Furthermore, the results revealed that the extraction methods studied in this work did not significantly affect the triple-helix structure of XCYPP.

### 3.8 In vitro chemical antioxidant activities of CYPP

**Fig. 7:**
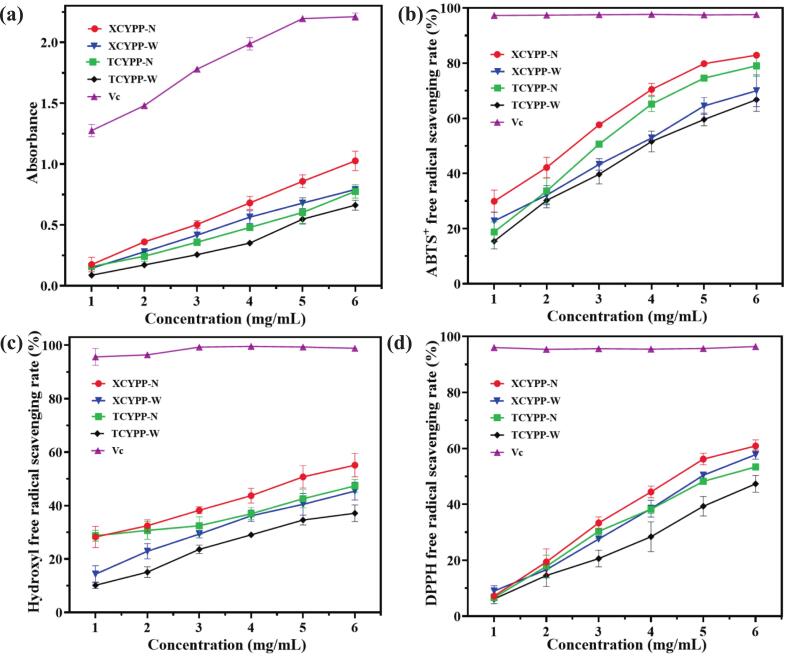
The in vitro antioxidant activities of CYPP. (a): total antioxidant capacity (T-AOC); (b): ABTS^+^free radical scavenging capacity; (c): hydroxyl free radical scavenging capacity; (d): DPPH free radical scavenging capacity.

It is reported that polysaccharides can scavenge various types of free radicals (Ding et al., 2025). To evaluate the antioxidant activities of the CYPP samples, the DPPH, ABTS^+^, and hydroxyl free radical scavenging capacities and T-AOC were investigated. It can be seen from Fig. 7 that the free radical scavenging capacities and T-AOC of CYPP increased gradually with increasing CYPP concentration in the range of 1–6 mg/mL. The DPPH free radical scavenging capacity and T-AOC of XCYPP regardless of extraction method were larger than those of TCYPP (Fig. 7(a)), which was probably due to the triple-helix structure of XCYPP and/or its high uronic acid content. The ultrasound-assisted NADES extraction method reduced the average *M*_w_ of XCYPP-N by cleaving long-chain polysaccharides into smaller fragments. The shorter polysaccharide chains exhibited lower steric hindrance, allowing more readily formation of intramolecular hydrogen bonds and hydrophobic interactions. However, the ABTS^+^ and hydroxyl free radical scavenging capacities of CYPP extracted by NADES regardless of *Chinese yam* variety were higher than those of CYPP extracted by water method (Fig. 7(b) and(c)). This phenomenon cannot be explained by triple-helix structure and uronic acid content. One possible explanation was that the CYPP extracted by NADES led to a formation of relatively compact structure, which might facilitate the enrichment of antioxidant active groups on its surface, enabling full exertion of ABTS^+^ and hydroxyl free radical scavenging capacities of TCYPP-N. The results suggested that a compact structure, triple-helix structure, and higher uronic acid content of CYPP could contribute to its antioxidant activity.

### 3.9 Effect of CYPP on protection of H_2_O_2_-induced HepG2 cell oxidative damage

**Fig. 8:**
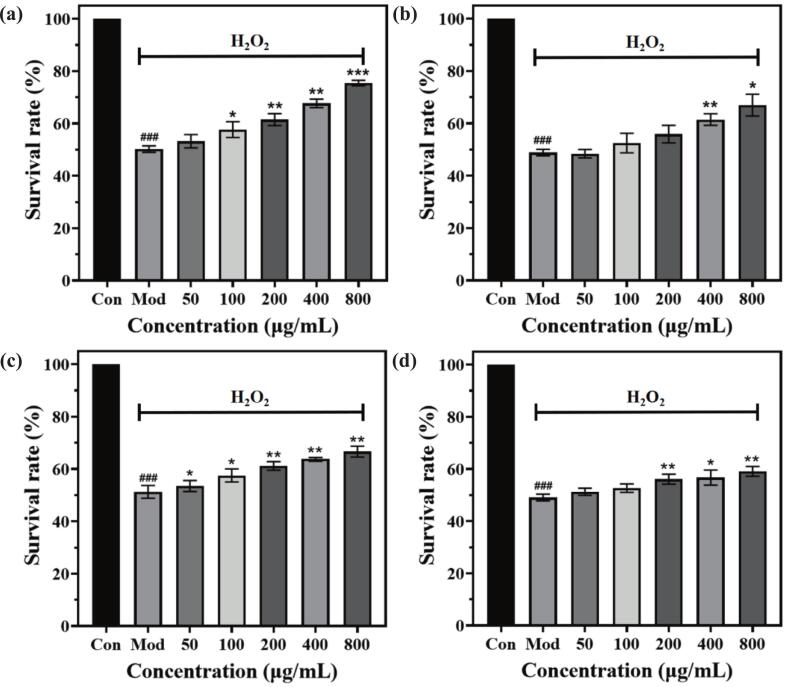
Effect of CYPP on the survival rate of HepG2 cells treated by H_2_O_2_. (a): XCYPP-N; (b): XCYPP-W; (c): TCYPP-N; (d): TCYPP-W. Note: ^###^*p* < 0.001 vs control group (0 μmol/L H_2_O_2_) is statistically significant; *p < 0.05, **p < 0.01, ***p < 0.001 vs model group (500 μmol/L H_2_O_2_) is statistically significant.

The HepG2 hepatocellular carcinoma cell line is widely employed as a cellular oxidative stress model (Liang et al., 2017). An H_2_O_2_-induced oxidative stress model was carried out and an H_2_O_2_ concentration of 500 μmol/L was selected as the optimal concentration for establishing the HepG2 cells oxidative stress model (Fig. S6). Furthermore, the cytotoxicity of four CYPP samples on HepG2 cells and MIHA cells was evaluated by using the MTT assay (Figs. S7 and S8). The results revealed that CYPPs exhibited no significant cytotoxicity toward HepG2 or MIHA cells. H_2_O_2_-induced HepG2 cells were selected as the oxidative damage model for subsequent experiments. The CYPP concentrations of 50, 100, 200, 400, and 800 μg/mL were selected for studying the protection effect against H_2_O_2_-induced HepG2 cell oxidative damage. It can be seen from Fig. 8 that compared with the control group, the model group (CYPP concentration of 0 μg/mL) treated with H₂O₂ at a concentration of 500 μmol/L exhibited a significant reduction in cell survival rate (^###^*p* < 0.001). In contrast, the intervention groups treated with CYPP significantly enhanced cell viability in a concentration-dependent manner in the range of 50–800 μg/mL. The results demonstrated that CYPP could not only scavenge free radicals, but also regulate intracellular oxidative balance. Notably, CYPP-N exhibited superior protective effects compared with CYPP-W. This may be due to the formation of a compact structure by the ultrasound-assisted NADES extraction method, which might enhance the interaction and/or permeability of the polysaccharide into the HepG2 cell membrane. However, a large-sample experiment is required to fully elucidate the structure-antioxidant activity relationships of CYPP.

## 4 Conclusions

In this study, a green and efficient method for extracting CYPP was established based on ultrasound-assisted NADES combined with alkaline proteinase deproteination. The extraction conditions were optimized as follows: molar ratio of BET to Urea was 1:2, water content was 40%, solid-liquid ratio was 1:40 g/mL, ultrasound temperature was 70 °C, and ultrasound time was 40 min. Under optimized extraction conditions, the highest extraction rate of CYPP-N was 150.30 ± 6.45 mg/g, which was 2.42-fold higher than that extracted by water method. Among the three physical factors (i.e., viscosity, pH, and polarity) of NADES, the viscosity of NADES exhibited a moderate negative correlation with the CYPP extraction rate and was the primary factor affecting the extraction rate. The FTIR result showed the presence of *β*-pyranose rings in all CYPP samples. XCYPP had a triple-helix structure and higher uronic acid content compared with TCYPP. Moreover, the results revealed that NADES extraction method did not affect the functional groups and triple-helix structure of CYPP, but increased the extraction rate, total saccharide content, Glc content, and uronic acid content of CYPP compared with the water extraction method. The AF4-MALS-dRI results indicated that NADES extracted CYPP had a relatively compact structure, which might contribute to scavenging free radicals and regulating intracellular oxidative balance. However, more comprehensive structural characterization and in vivo assays are needed to fully elucidate the structure-antioxidant activity relationships of CYPP.

## CRediT authorship contribution statement

**Yao Huang:** Writing – original draft, Software, Investigation. **Liu Yang:** Writing – original draft, Investigation, Data curation. **Tinghui Yin:** Formal analysis. **Mu Wang:** Visualization, Validation. **Siyu Wang:** Project administration, Methodology. **Weiming Wang:** Writing – review & editing, Resources. **Haiyang Dou:** Writing – review & editing, Supervision, Funding acquisition, Conceptualization.

## Declaration of competing interest

The authors declare that they have no known competing financial interests or personal relationships that could have appeared to influence the work reported in this paper.

## Data Availability

Data will be made available on request.
