# Supplementary material for: A comprehensive study on the relationship between structure and antioxidant activity of natural deep eutectic solvent extracted polysaccharides from Chinese yam peel using asymmetrical flow field-flow fractionation
Source: Food Chem X. 2026 Jul 2;38:104167. doi: 10.1016/j.fochx.2026.104167 (PMC13425809; doi:10.1016/j.fochx.2026.104167)
Supplement: Supplementary material [file mmc1.docx]

A comprehensive study on the relationship between structure and antioxidant activity of natural deep eutectic solvent extracted polysaccharides from *Chinese yam* peel using asymmetrical flow field-flow fractionation

Yao Huang^a,#^, Liu Yang^a,#^, Tinghui Yin^a^, Mu Wang^a^, Siyu Wang^a^, Weiming Wang^b,^*, Haiyang Dou^a,c,d,^*

^a^*Key Laboratory of Pathogenesis Mechanism and Control of Inflammatory-Autoimmune Disease of Hebei Province, School of Basic Medical Sciences, Hebei University, Baoding 071000, China*

^b^*State Key Laboratory of New Pharmaceutical Preparations and Excipients, Hebei University, Baoding 071002, China*

^c^*Key Laboratory of Medicinal Chemistry and Molecular Diagnosis of Ministry of Education, Hebei University, Baoding 071002, China*

Tel: 86-312-507-5532

Fax: 86-312-593-7102

*Corresponding authors E-mail: [rurenlong@163.com](mailto:rurenlong@163.com) (W. Wang) and douhaiyang-1984@163.com (H. Dou)

#These authors contributed equally to this work.


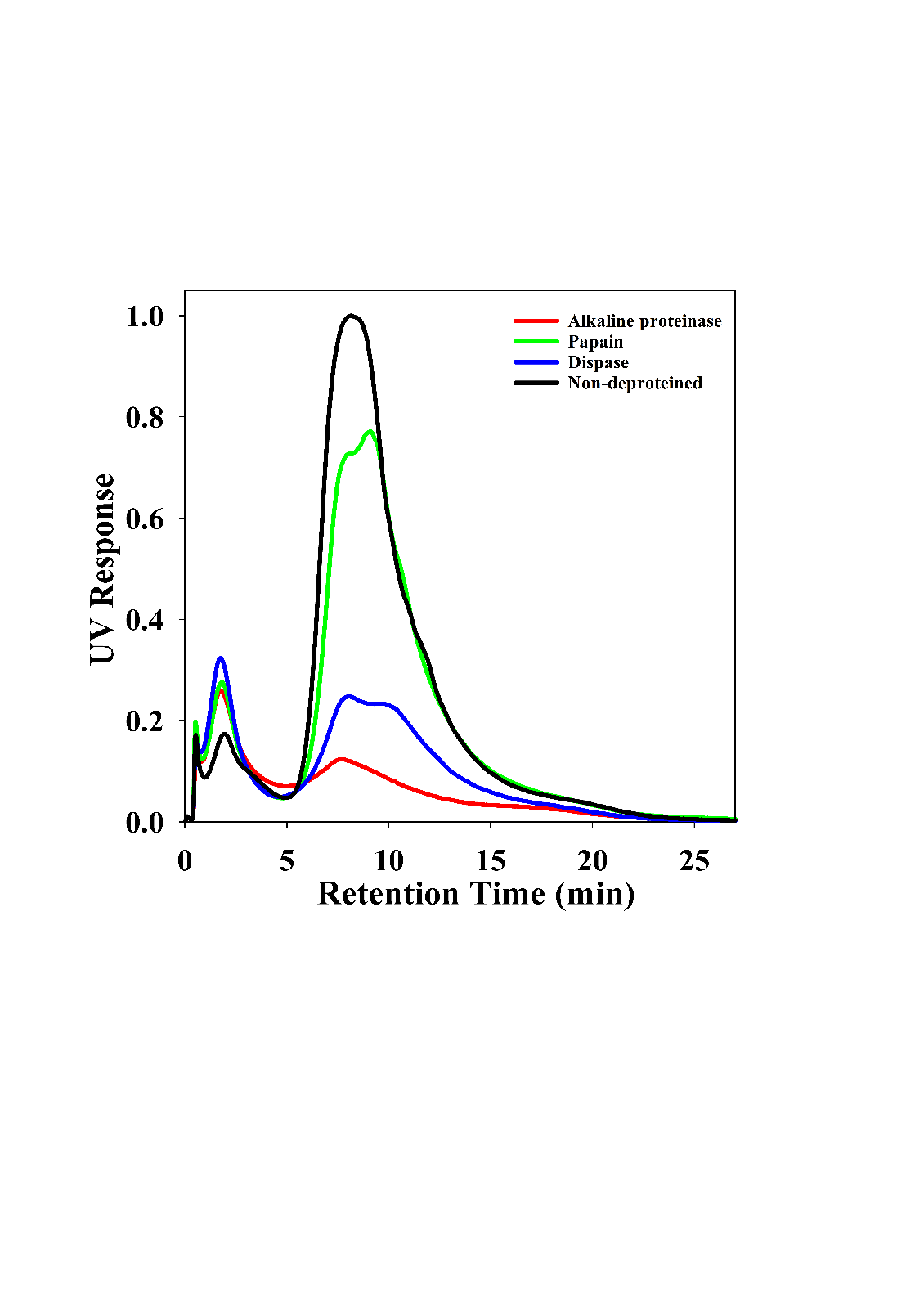


Fig. S1 AF4-UV fractograms of CYPP treated by different types of proteases.

The channel flow rate was 1 mL/min and crossflow rate started at 2.0 mL/min and decreased exponentially to 0.1 mL/min with a half-life of 1.8 min. Carrier liquid was deionized water containing 5 mM NaNO_2_ (pH 7.0), and the injection volume was 100 µL with a concentration of 1 mg/mL. UV detector was operated at wavelength of 280 nm.

(a)
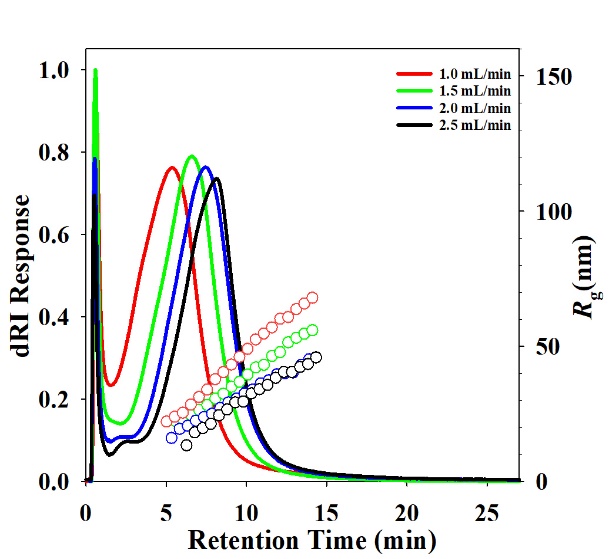
(b)
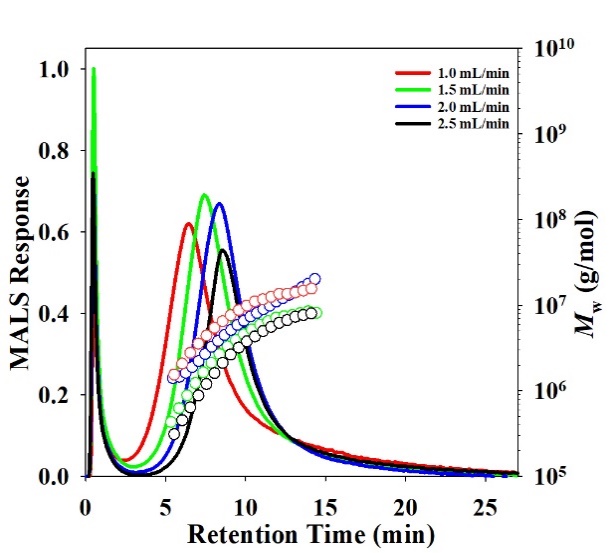


(c)
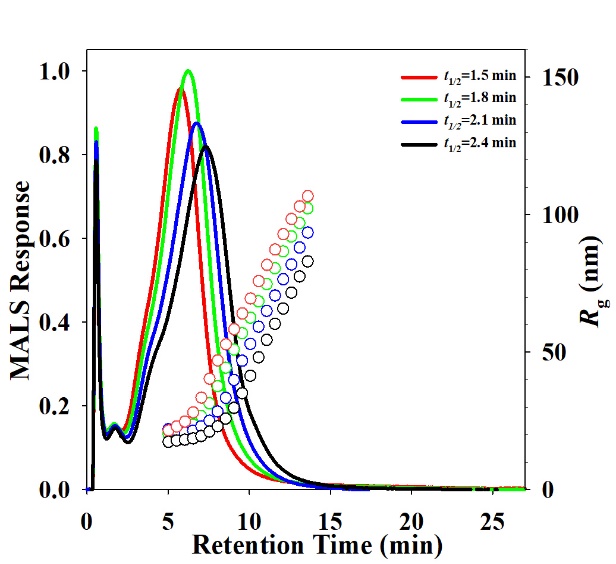
(d)
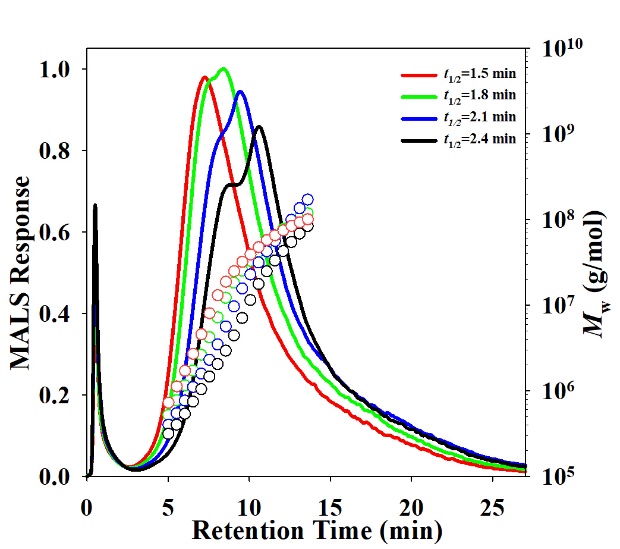


Fig. S2 AF4-MALS-dRI fractograms, *R*_g_, and *M*_w_ distributions of CYPP obtained with different cross flow rates (a, b) and half-life time (c, d).

Carrier liquid was deionized water containing 5 mM NaNO_2_ (pH 7.0) and the sample injection volume was 100 µL with a concentration of 1 mg/mL.


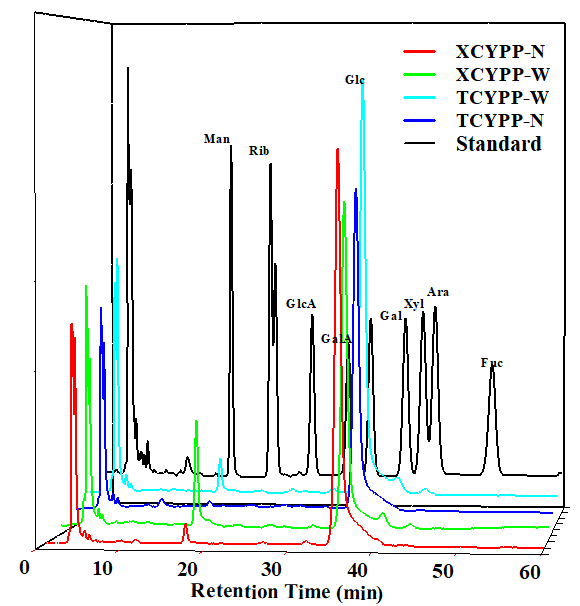


Fig. S3 Monosaccharide composition of CYPPs.

The flow rate was 1 mL/min. The mobile phase consisted of acetonitrile and monopotassium phosphate-NaOH buffer solution (pH 6.7, 0.05 M) at the ratio of 16: 84 (*v/v*), and UV detector was operated at wavelength of 250 nm.


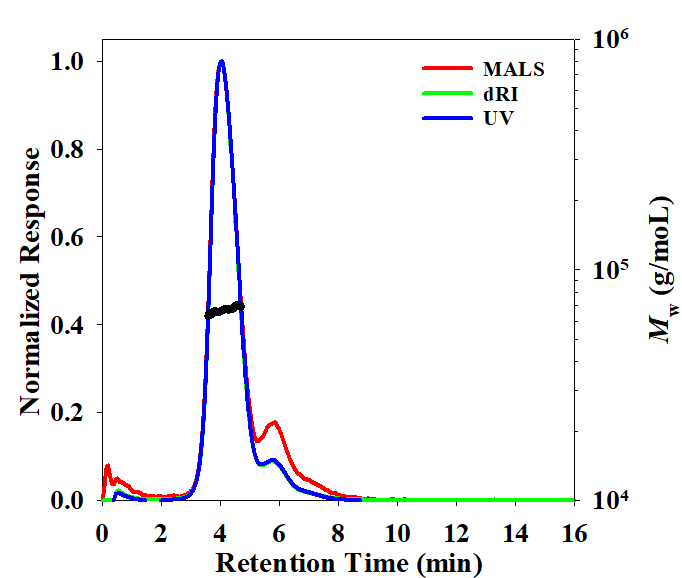


Fig. S4 AF4-MALS-dRI-UV fractograms of BSA.

The channel flow and cross flow rate were 1 mL/min and 3 mL/min, respectively. Carrier liquid was deionized water containing 5 mM NaNO_2_ (pH). The injection volume was 100 µL with a concentration of 1 mg/mL, and UV detector was operated at wavelength of 280 nm.


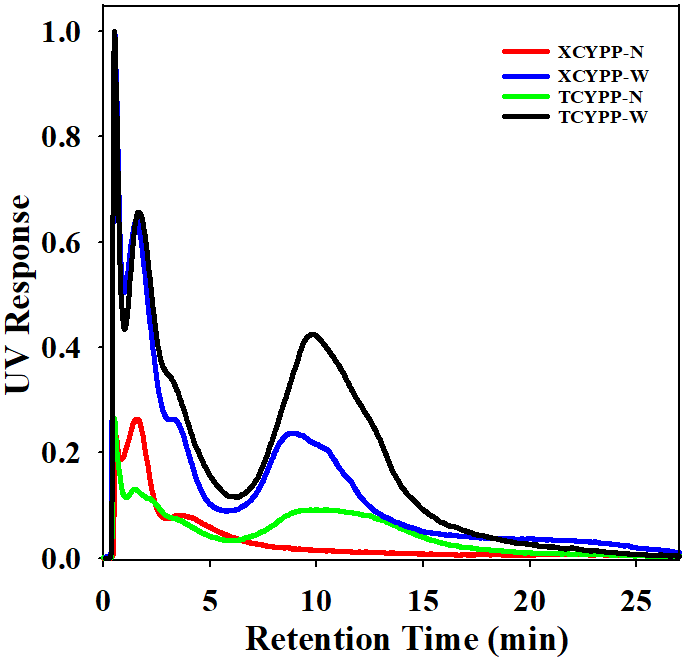


Fig. S5 AF4-UV fractograms of CYPPs.

The channel flow rate was 1 mL/min and the crossflow rate started at 2.0 mL/min and decreased exponentially to 0.1 mL/min with a half-life of 1.8 min. Carrier liquid was deionized water containing 5 mM NaNO_2_ (pH 7.0), and the injection volume was 100 µL with a concentration of 1 mg/mL. UV detector was operated at wavelength of 280 nm.


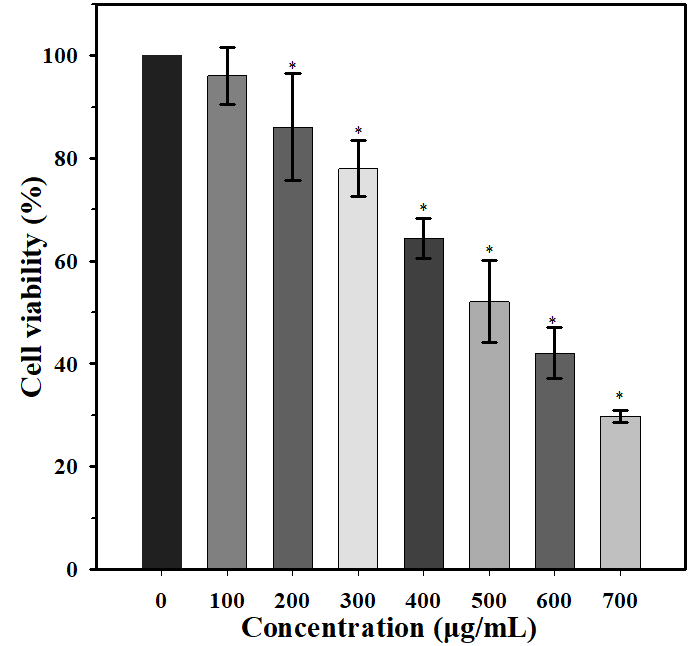


Fig. S6 Effect of concentration of H_2_O_2_ on the viability of HepG2 cells.

Note: **p* < 0.05 *vs* control (0 μmol/L H_2_O_2_) is statistically significant.

(a)
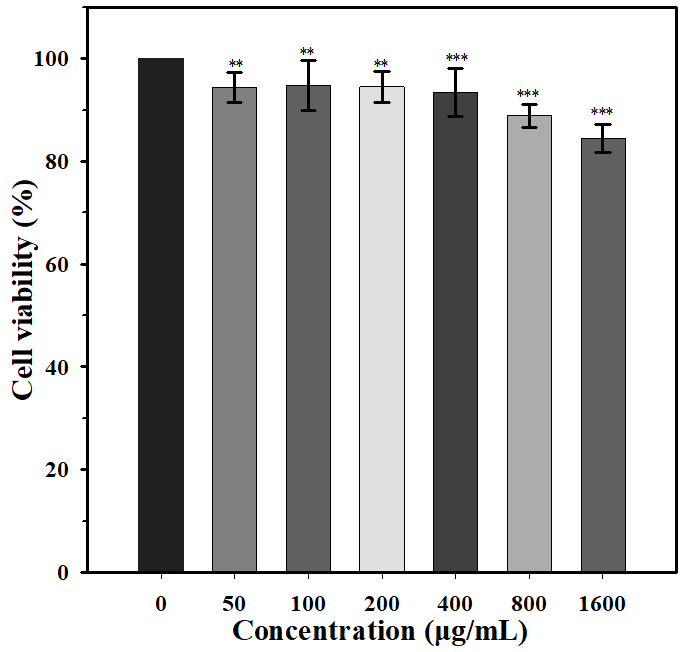
 (b)
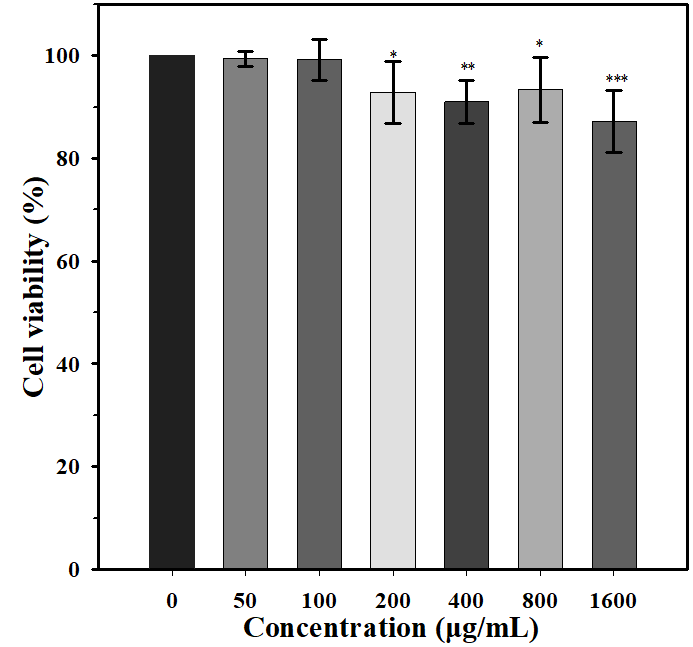


(c)
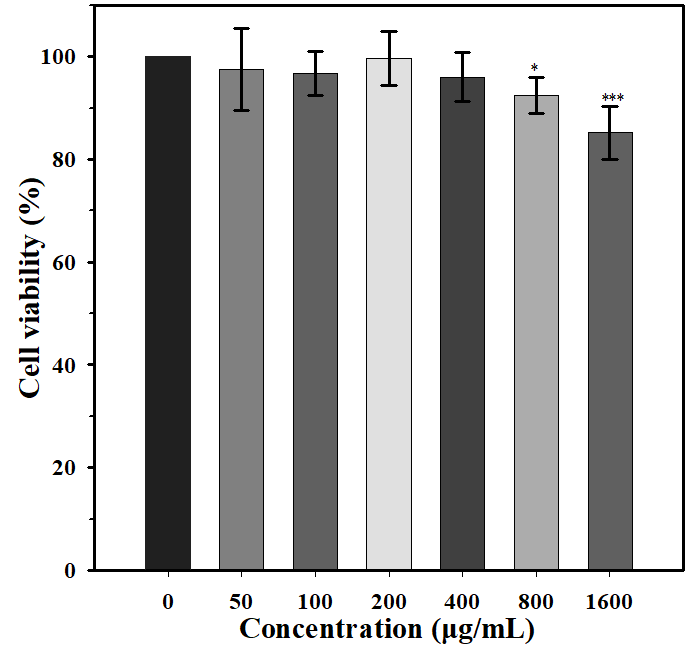
 (d)
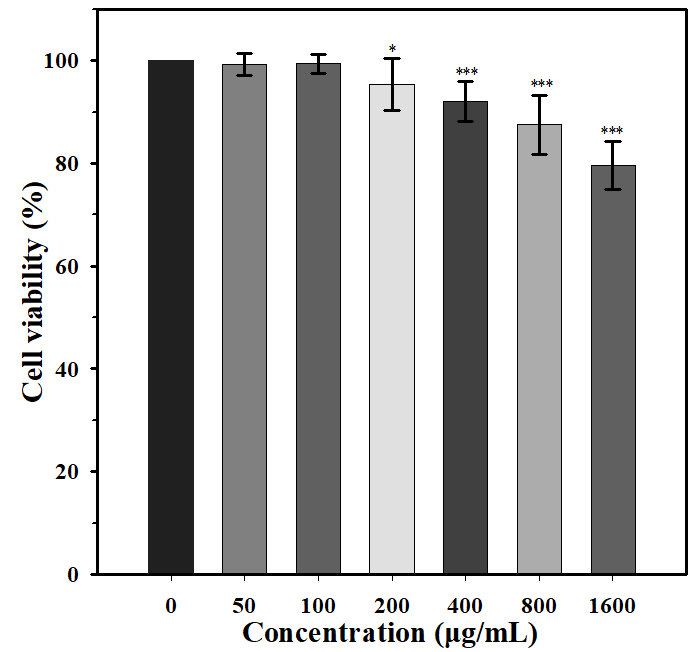


Fig. S7 Effect of CYPP on the viability of HepG2 cells.

(a) XCYPP-N; (b) XCYPP-W; (c) TCYPP-N; (d) TCYPP-W

Note: **p*<0.05, ***p* <0.01, ****p*<0.001 *vs* model group (500 μmol/L H_2_O_2_) is statistically significant.

(a)
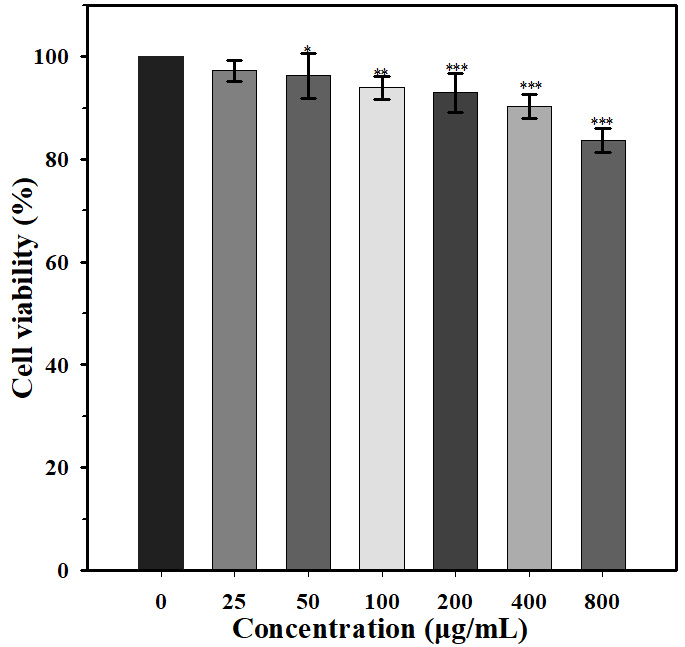
 (b)
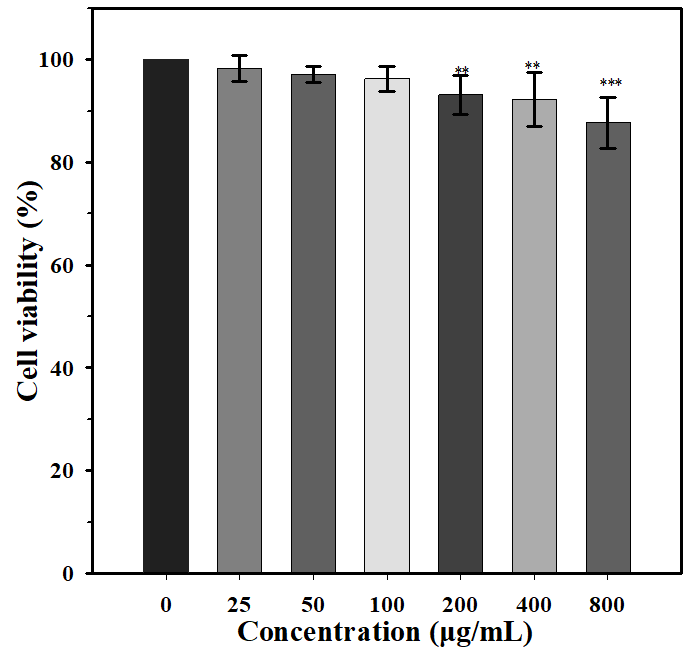


(c)
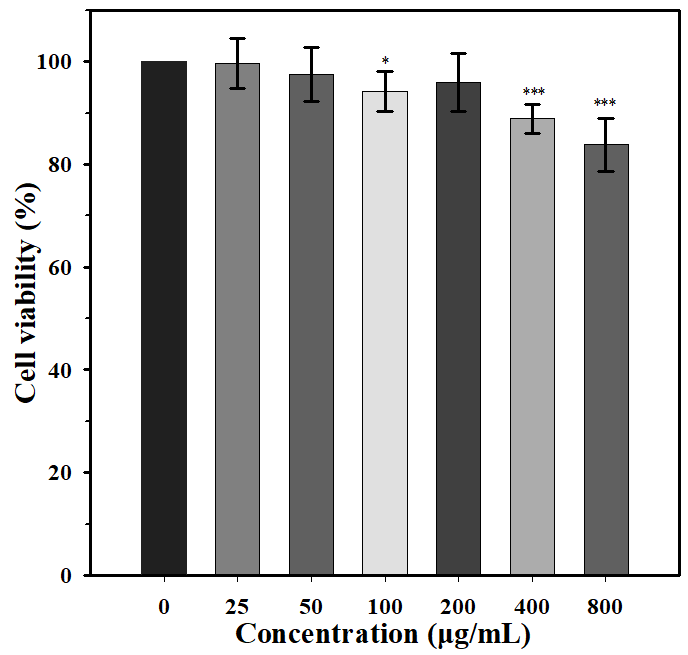
 (d)
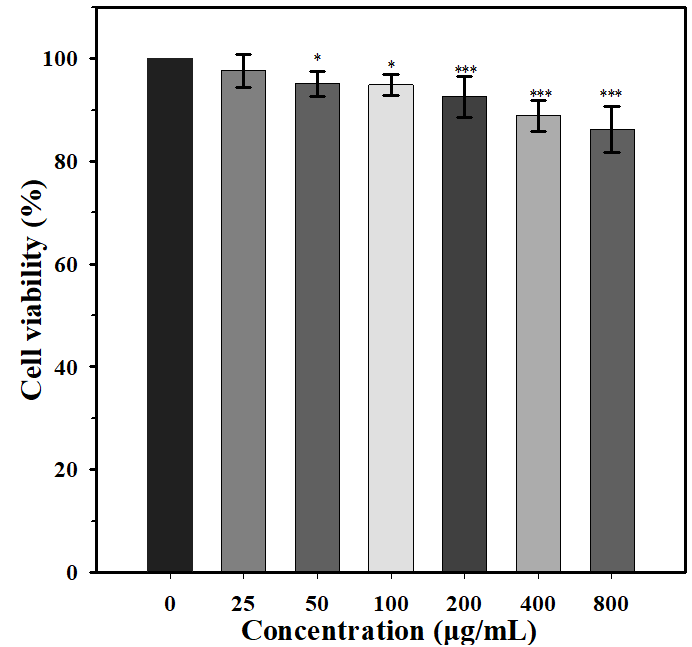


Fig. S8 Effect of CYPP on the viability of MIHA cells treated by H_2_O_2_.

(a) XCYPP-N; (b) XCYPP-W; (c) TCYPP-N; (d) TCYPP-W

Note: **p*<0.05, ***p* <0.01, ****p*<0.001 *vs* model group (500 μmol/L H_2_O_2_) is statistically significant.
